# Impact of Morphine on Early Recovery After Anterior Cruciate Ligament Reconstruction: A Case-Control Study

**DOI:** 10.7759/cureus.117127

**Published:** 2026-09-29

**Authors:** Mohamad K Moussa, Fethi Mrabet, Eugénie Valentin, Olivier Grimaud, Frederic Khiami, Antoine Gerometta, Alain Meyer, Nicolas Lefevre, Alexandre Hardy

**Affiliations:** 1 Orthopedic Surgery, Clinique du Sport, Paris, FRA; 2 Orthopaedic Surgery, Clinique du Sport, Paris, FRA

**Keywords:** acl reconstruction, morphine, postoperative pain, rehabilitation, sports medicine

## Abstract

Purpose: Effective pain management is crucial in postoperative recovery after Anterior Cruciate Ligament Reconstruction (ACLR). To examine the effectiveness of the new opioid-containing protocol compared to another previously published protocol based on Nefopam and dynamic compression cryotherapy.

Methods: This retrospective analysis of prospectively collected data was conducted at a sports surgery center between 2023 and 2024, including 1116 patients undergoing primary isolated ACLR. Patients were divided into two matched groups based on postoperative pain management: the opioid group (N=556), treated with oral morphine sulfate (Actiskenan®) as needed in addition to multimodal analgesia (e.g., paracetamol, nefopam, and ketoprofen), and the non-opioid group N=556, treated with multimodal analgesia alone. Patients were matched for age, sex, BMI, Lysholm score, Tegner score, meniscal injury, and chondropathy. The primary outcome was pain on the visual analogue scale (VAS) in the early postoperative period, analyzed in relation to the minimal clinically important difference (MCID) and the patient acceptable symptom state (PASS) thresholds for VAS. The secondary outcome was side effects, including nausea, dizziness, vomiting, anxiety, and abdominal pain, as well as early complications and revision rate.

Results: The mean age in both groups was comparable (32.2 ± 9.4 years in the non-opioid group [N = 556] and 32.5 ± 10.0 years in the opioid group [N = 556], p = 0.867). Both groups predominantly used the quadrupled hamstring graft technique (440/556, 80.0% vs. 438/556, 79.9%, p = 0.4455). Preoperative scores were similar (p > 0.05). Postoperative pain was higher in the non-opioid group: day 0 VAS scores were 5.6 ± 2.7 (n = 536/556) vs. 5.3 ± 2.7 (n = 522/556) (p = 0.0339), and night pain was more common (379/556, 68.2% vs. 341/556, 61.3%, p = 0.0202). This trend continued on day 1 (5.2 ± 2.4 [n = 545/556] vs. 4.8 ± 2.4 [n = 543/556], p = 0.0014), day 2 (4.5 ± 2.4 [n = 551/556] vs. 4.2 ± 2.3 [n = 550/556], p = 0.0182), and day 3 (3.8 ± 2.3 [n = 551/556] vs. 3.4 ± 2.2 [n = 551/556], p = 0.0080). The non-opioid group had higher rates of dizziness on day 0 (100/536, 18.7% vs. 70/522, 13.4%, p = 0.0236). There was a trend toward higher complications in the non-opioid group (11/556, 2.0% vs. 3/556, 0.5%, p = 0.0558), mainly due to Cyclops syndrome and knee stiffness. Reoperation rates were similar (p > 0.05).

Conclusion: Adding morphine to the postoperative protocol was associated with reduced postoperative pain in the first 3 days postoperatively and dizziness on the first day. However, while statistically significant, the mean difference in VAS scores did not exceed the MCID.

## Introduction

The rupture of the anterior cruciate ligament is among the most common injuries in knee traumatology, particularly among athletes [1]. It can lead to significant instability, which may hinder the resumption of sports activities and may potentially evolve into secondary osteoarthritis [1,2]. Surgical anterior cruciate ligament reconstruction (ACLR) is predominantly at the forefront of the treatment strategy and is associated with moderate to severe pain ranging from 2.5 to 5.4 as evaluated by the visual analogue scale (VAS) [3,4]. Post-operative pain can reduce the functional performance of the knee joint, prolong the duration of hospital stays, and even affect the patient’s outcomes [5,6].

Accurate postoperative pain management is essential and can significantly reduce the costs of care associated with outpatient surgery [7]. Despite the wide variety of analgesics used, the optimal protocol associated with the fastest recovery and least side effects profile is yet to be identified [8]. Ongoing studies on the subject assess different protocols in light of the VAS using multiple approaches. This includes the assessment of the minimally clinically important difference (MCID) and the patient acceptable symptom state (PASS) [9-14]. The PASS denotes the threshold at which patients deem their condition to be satisfactory and manageable post-treatment. The standard for PASS in relation to the VAS typically ranges from 3.4 to 4 [9,13-15]. The MCID represents the smallest change in a condition that patients perceive as beneficial and is generally considered to be between 1 and 1.4 on the VAS, subject to the specific research [10-12,16].

Oral morphine with rapid release is a formulation of morphine sulfate commonly used as a standard treatment against acute pain, such as after ACL surgery, due to its moderate to strong analgesic properties. However, despite the well-known pharmacokinetic profile of this molecule and its wide use in the management of postoperative pain, its benefits must be balanced with the risk of increasing the dependence potential [17-21].

In a recent investigation, Moussa et al. explored the efficacy of two post-operative pain management protocols following ACLR [16]. Their study revealed that a dynamic intermittent compression cryotherapy protocol paired with intravenous nefopam expedited pain recovery by one day compared to a control group using static compression cryotherapy with oral nefopam [16]. Notably, their protocol did not include morphine, and although it proved the superiority of dynamic techniques over static ones, the authors acknowledged that the VAS scores in their study were marginally higher than those commonly cited in the literature. This discrepancy set the stage for the current study, which aims to evaluate the enhanced protocol from Moussa et al.’s research against a similar regimen that incorporates morphine.

The primary objective of this study was to compare early postoperative pain (VAS scores evaluated against the MCID) following primary ACL reconstruction between two multimodal analgesic pathways: an oral morphine-containing protocol and an intravenous nefopam-based protocol. The secondary objectives were to compare early recovery trajectories (defined by the proportion of patients achieving the PASS threshold), the incidence of postoperative adverse effects, and 90-day early complications and reoperation rates. We hypothesized that the morphine-containing multimodal protocol would be associated with improved early pain relief and accelerated attainment of the PASS threshold without increasing postoperative adverse events.

## Materials and methods

Study design and inclusions

This case-control study was a retrospective analysis of prospectively data conducted at a sports surgery referral center between 2023 and 2024. Both cohorts were recruited concurrently during this two-year timeframe and do not represent a historical control from our previous publication. The study was approved by the institutional review board (CPP IDF IV), and written consent was obtained from all patients.

Inclusion criteria included all adult patients >18-years-old treated with primary isolated ACLR. Exclusion criteria included revision surgery, multi-ligamentous injury, bony avulsion injury, and patients who refused to be enrolled in the study.

Patients were categorized into two distinct postoperative multimodal analgesia pathways: an oral morphine-containing protocol (incorporating oral morphine sulfate rescue without nefopam) and a non-opioid multimodal protocol (utilizing continuous intravenous nefopam infusion paired with metoclopramide).

Analgesia protocol

All patients underwent surgery under spinal anesthesia in combination with ultrasound-guided saphenous nerve block using 40 mg of ropivacaine, and peri-articular infiltration with ropivacaine (1 vial of 2%). In the immediate postoperative period, patients received intermittent compression cryotherapy using CryoNov®, positioned by the surgeon in the operating room. The device was set at low pressure with a 30-minute frequency, removed at night, and kept for 5 days.

The standard pain relief protocol lasted 8 days and included 200 mg of oral Celecoxib twice daily, Lamaline (Paracetamol/opium) 500 mg/25 mg every 8 hours, Omeprazole 20 mg/day, and Paracetamol 500 mg every 8 hours.

The non-opioid group received intravenous administration of three vials of Nefopam 20 mg/2 mL diluted in 50 mL of normal saline solution (9%) as a continuous IV for 12 hours, along with one vial of IV metoclopramide 10 mg/2 mL diluted in 50 mL of normal saline for 30 minutes every 12 hours. In the opioid group, Nefopam was replaced by morphine Actiskenan at 10mg/6 hours as needed. Table 1 summarizes both analgesia protocols.

**Table 1:** Analgesia protocol in both groups.

Protocol Component	Opioid Protocol Group	Non-Opioid Protocol Group
Anesthesia	Spinal anesthesia with saphenous nerve block (40 mg ropivacaine)	Spinal anesthesia with saphenous nerve block (40 mg ropivacaine)
Peri-articular Infiltration	Ropivacaine (1 vial of 2%)	Ropivacaine (1 vial of 2%)
Postoperative Cryotherapy	CryoNov (intermittent compression, low pressure, 30-min frequency, removed at night, for 5 days)	CryoNov (intermittent compression, low pressure, 30-min frequency, removed at night, for 5 days)
Scheduled Analgesia (8 Days)	Celecoxib 200 mg orally twice daily Lamaline (Paracetamol/opium) 500 mg/25 mg every 8 hours Omeprazole 20 mg daily Paracetamol 500 mg every 8 hours	Celecoxib 200 mg orally twice daily Omeprazole 20 mg daily Paracetamol 500 mg every 8 hours
Nefopam Infusion	Not included	IV administration of 20 mg/2 ml diluted in 50 ml normal saline (0.9%), continuous IV over 12 hours every 12 hours
Metoclopramide Infusion	Not included	IV administration of 10 mg/2 ml diluted in 50 ml normal saline over 30 minutes every 12 hours
Rescue Opioid	Oral morphine sulfate (Actiskenan) 10 mg every 6 hours as needed	Not included

Surgical technique and postoperative rehabilitation protocol 

All patients were treated by senior orthopedic surgeons AH, NL, YB, AG, OG, AM. Several techniques were utilized. These included hamstring grafts (such as quadrupled semitendinosus, doubled gracilis, and doubled semitendinosus), the biofast technique, with or without a lateral extraarticular procedure, bone-patellar-tendon-bone graft, and fascia lata graft.

Outcome measures

The primary outcome measure was the VAS [22] in the early post-operative period, compared between both groups. This included assessments on the night of surgery (Day 0), as well as postoperative days 1, 2, and 3 (Appendix, Table 7-10). The study analyzed this outcome in light of the MCID for VAS, which is set in the literature between 1 and 1.4 [10-13].

Secondary outcome measures included the rate of patients who crossed the PASS for VAS at each time point. The PASS for VAS was set between 3.2 and 4 [13,15]. The most substantial values reported for the MCID (1.4) and the minimal values for the PASS (3.2) were selected for use in our analysis to ensure a more conservative comparison.

Other outcome measures include the rate of side effects as compared between the groups. This includes nausea, dizziness, vomiting, anxiety, and abdominal pain.

Data collection

Data were prospectively collected using the online software Websurvey®, which is dedicated to research. Surgeons accessed it to enter history, physical examination, and surgical details, while patients used it to input demographic information and clinical outcomes. At every study checkpoint (days 0, 1, 2, 3), patients received notifications through email and phone messages, reminding them to assess and report their pain using the VAS. This approach guaranteed that responses were objective and not swayed by outside factors.

Participants and sample size

During the study timeframe, 2086 patients who underwent ACL reconstruction surgery completed a questionnaire regarding their pain during the first three days post-operatively. Of them, 586 were excluded for the following reasons: 221 patients were younger than 18-years old, 126 patients had multiligamentous injury, and 239 had missing data.

The number of eligible patients for matching was 558 in the opioid group and 942 in the non-opioid group. Patients were matched for age, sex, BMI, Lysholm score, Tegner score and meniscal injury, chondral lesion producing 2 groups: 556 patients in the opioid group and 556 patients in the non-opioid group (Figure 1).

**Figure 1:**
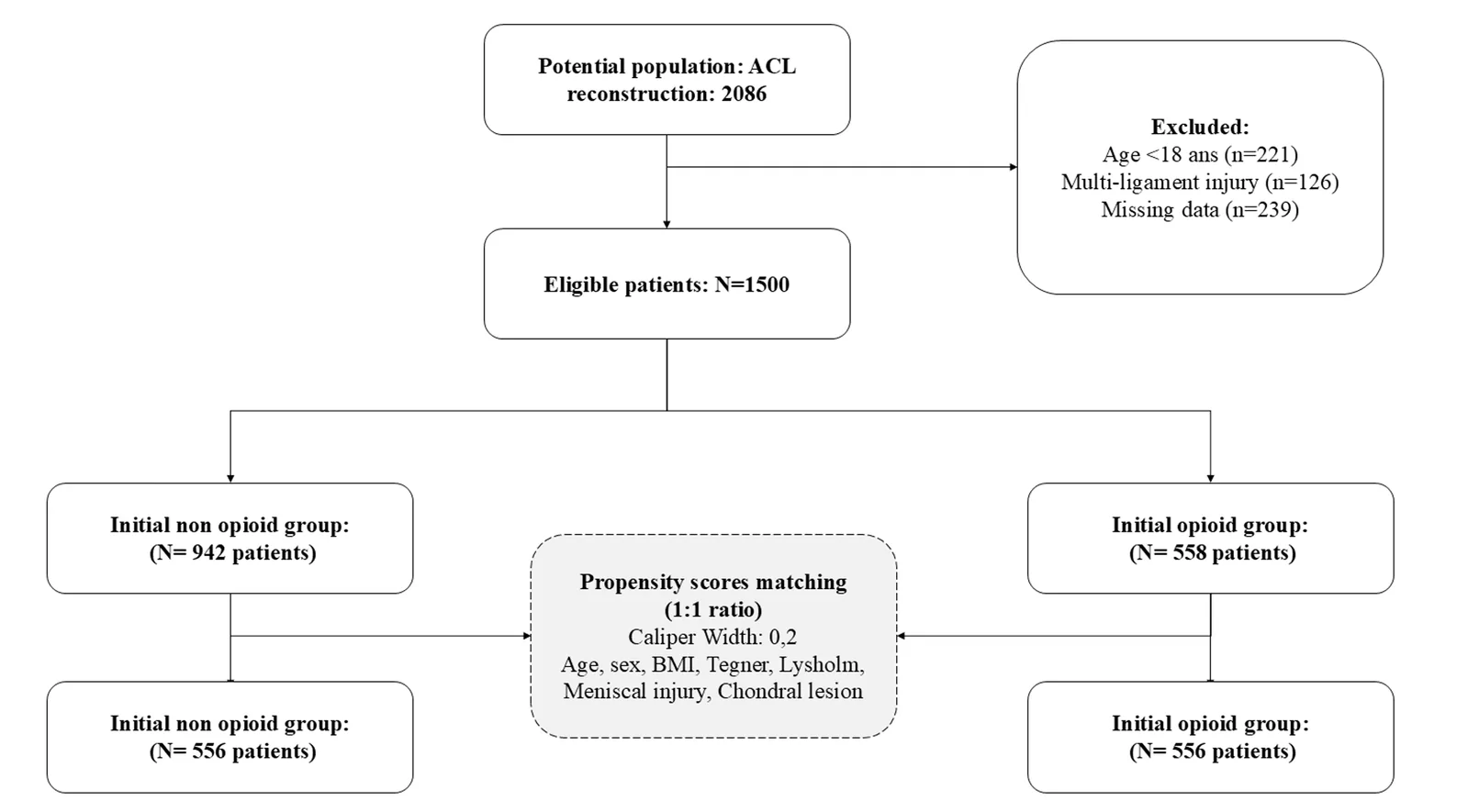
Flow chart ACL: Anterior cruciate ligament BMI: Body mass index

Statistical analysis

The study population was matched using a propensity score to reduce selection bias between the two groups. Participants were matched in a 1:1 ratio, with the matching process performed on a logit scale with a caliper width of 0.2. The variables used for matching included age, sex, Tegner score [23], Lysholm score [24], the presence of meniscal injury and chondropathy, and BMI. Qualitative variables were described by their frequency and percentage, and comparisons were made using the Pearson chi-square test or Fisher's exact test, depending on group sizes.

Propensity score matching was performed in R software (Version 4.2.0; R Foundation for Statistical Computing, Vienna, Austria) using the MatchIt package with nearest-neighbor 1:1 matching without replacement on the logit scale (caliper = 0.2 standard deviations of the logit). Post-matching balance was confirmed via standardized mean differences (SMD < 0.1 across all covariates). Patients completed online VAS assessments within standardized 4-hour notification windows (evening assessment between 18:00 and 22:00, morning/daily between 08:00 and 12:00). Missing data were managed via complete-case analysis for primary endpoints, with missing frequencies reported in Table 5; sensitivity analyses using multiple imputation yielded concordant results.

Quantitative variables were described using means and standard deviations and were compared using the appropriate statistical tests, either the Student's t-test or the Mann-Whitney U test, based on the data distribution. A P-value of 0.05 was considered statistically significant. All statistical analyses were conducted using R software (Version 4.2; R Foundation for Statistical Computing).

## Results

Demographic characteristics

The mean age in both groups was comparable (32.2 ± 9.4 years in the non-opioid group and 32.5 ± 10.0 years in the opioid group, p = 0.867). The delay to surgery was 13.1 ± 38.2 months in the non-opioid group and 16.9 ± 47.2 months in the opioid group (p = 0.1103). The techniques utilized were similar across both groups, with the most utilized technique being the quadrupled hamstring graft, which was performed in 440 out of 556 patients (80.0%) in the non-opioid group and 438 out of 556 patients (79.9%) in the opioid group (p = 0.4455) (Table 2).

**Table 2:** Demographic characteristics of patients. Data are presented as mean ± standard deviation (SD) for continuous variables, and as frequencies n (%) for categorical variables.

Demographic characteristics of the two groups	Non opioid group	Opioid group	p-value
	N=556	N=556	
Age, mean (SD)	32.2 (9.4)	32.5 (10.0)	0.867
Sex, N(%)			
Female	248 (44.6%)	248 (44.6%)	>0.9999
Male	308 (55.4%)	308 (55.4%)	
Body mass index (BMI), Mean (SD)	23.8 (3.2)	23.8 (3.4)	0.501
Operating side, N(%)			
Right	268 (48.2%)	265 (47.7%)	0.9044
Left	288 (51.8%)	291 (52.3%)	
Type of hospitalization, N(%)			
Ambulatory	533 (96.6%)	535 (96.7%)	0.8691
Classic	19 (3.4%)	18 (3.3%)	
Unknown	4 (0.7% of the total)	3 (0.5% of the total)	
Sports practice, N(%)			
Competition	211 (37.9%)	180 (32.3%)	0.3593
occasional practice (active)	61 (11.0%)	62 (11.2%)	
Leisure sport activity (regular practice)	252 (45.3%)	282 (50.8%)	
professional	25 (4.5%)	25 (4.5%)	
Sedentary	7 (1.3%)	7 (1.3%)	
Type of sport, N(%)			
No sports activity	6 (1.1%)	10 (1.8%)	0.763
pivot shift (foot, hand, rugby, basket, judo)	295 (53.1%)	299 (53.8%)	
Without Pivot shift (tennis, ski,badminton,volley..)	152 (27.3%)	150 (27.0%)	
without pivot (jogging, bike, swimming)	103 (18.5%)	97 (17.4%)	
Circumstance of the accident, N(%)			
Public Road traffic accident	20 (3.6%)	18 (3.2%)	0.9796
Accident at work (Work injury)	23 (4.1%)	25 (4.5%)	
Domestic accident injury	17 (3.1%)	16 (2.9%)	
Sports injuries	496 (89.2%)	497 (89.4%)	
Meniscal injury, N(%)			
No	335 (60.3%)	335 (60.3%)	>0.9999
Yes	221 (39.7%)	221 (39.7%)	
If Yes,			0.844
External meniscus injury, N(%)	96 (17.3%)	99 (17.8%)	>0.9999
Internal meniscus injury, N(%)	160 (28.8%)	160 (28.8%)	
Chondropathy, N(%)			
No	483 (86.9%)	483 (86.9%)	>0.9999
Yes	73 (13.1%)	73 (13.1%)	
Type of graft ACLR, N(%)			
Allograft	1 (0.2%)	0 (0.0%)	0.4455
Quadrupled hamstring graft	440 (80.0%)	438 (79.9%)	
Bone-patellar tendon- bone graft	18 (3.2%)	11 (2.0%)	
Modified Macintosh	91 (16.5%)	99 (18.1%)	
Unknown	6 (1% of the total)	8 (1.4% of the total)	
Accident/surgery delay in months, mean (sd)	13.1 (38.2)	16.9 (47.2)	0.1103
Lysholm score pre-op, mean (sd)	72.4 (16.5)	72.6 (16.8)	0.6528
Tegner score before injury, mean (sd)	6.9 (1.9)	6.9 (1.9)	0.8808

Pain comparison

The non-opioid group reported higher pain scores compared to the opioid group, with the largest difference observed on the night of day 0 (5.7 vs. 5.2, p = 0.0023). Higher pain levels in the non-opioid group persisted across subsequent days. However, these differences did not exceed the MCID threshold (Table 3).

**Table 3:** Pain comparisons between groups. Data are presented as mean ± standard deviation (SD). VAS: Visual Analogue Scale

Pain Comparison	Non-opioid Group (N=556)	Opioid Group (N=556)	p-value
VAS Day 0, mean (SD)	5.6 (2.7)	5.3 (2.7)	0.0339
VAS Night Day 0, mean (SD)	5.7 (2.5)	5.2 (2.6)	0.0023
Night Pain Day 0, N (%)			
- No	177 (31.8%)	215 (38.7%)	0.0202
- Yes	379 (68.2%)	341 (61.3%)	
VAS Day 1, mean (SD)	5.2 (2.4)	4.8 (2.4)	0.0014
VAS Day 2, mean (SD)	4.5 (2.4)	4.2 (2.3)	0.0182
VAS Day 3, mean (SD)	3.8 (2.3)	3.4 (2.2)	0.008

Result of PASS

In addition to evaluating overall pain levels, we also examined how many patients reached the PASS for the VAS score, using a threshold set at 3.2. Overall, a greater proportion of patients in the opioid group reached this threshold at every time point compared to the non-opioid group. On the day of surgery, 156 out of 556 patients (28.1%) in the opioid group reported satisfactory pain levels, versus 143 out of 556 patients (25.7%) in the non-opioid group (p = 0.42). During the first postoperative night, 155 out of 556 patients (27.9%) in the opioid group achieved the PASS, compared to 129 out of 556 patients (23.2%) in the non-opioid group (p = 0.09).

Notably, the difference became statistically significant starting on day 1, with 33.8% (n=188/556) of opioid-treated patients reaching the PASS compared to 28.0% (n=156/556) in the non-opioid group (p = 0.04). This trend persisted: by day 2, 42.8% (n=238/556) of the opioid group had achieved acceptable symptom states versus 35.6% (n=198/556) in the non-opioid group (p = 0.02), and by day 3, the gap widened further (55.9%, n=311/556 vs. 46.9%, n=261/556; p < 0.01).

Sensitivity analysis applying alternative PASS cutoffs demonstrated consistent results: utilizing a threshold of 3.4, achievement rates favored the opioid group on Day 1 (35.6% vs. 29.7%, p = 0.038) and Day 3 (58.1% vs. 49.6%, p = 0.005). When evaluating a threshold of 4.0, significantly higher attainment persisted in the opioid cohort across Day 1 (43.9% vs. 36.5%, p = 0.012), Day 2 (54.0% vs. 46.8%, p = 0.017), and Day 3 (65.6% vs. 57.2%, p = 0.004), confirming the stability of our primary PASS findings across different criteria (Table 4).

**Table 4:** Percentage of patients crossing the PASS for VAS (3.2 threshold) Data are presented as frequencies n (%).

	Non opioid group	Opioid group	p-value
Day 0	143(25.7%)	156(28.1%)	0.42
First night	129(23.2%)	155(27.9%)	0.09
Day 1	156(28.0%)	188(33.8%)	0.04
Day 2	197(35.6%)	238(42.8%)	0.02
Day 3	261 (46.9%)	311(55.9%)	< 0.01

Side effects

The non-opioid group experienced significantly higher dizziness on day 0 compared to the opioid group (18.7%, n=104/556 vs. 13.4%, n=75/556; p = 0.0236). No other side effects showed significant differences between the groups across all days (Table 5).

**Table 5:** Side effects of analgesia protocol in both groups. Data are presented as frequencies n (%).

Side effects	Non opioid group	Opioid group	p-value
	N=556	N=556	
Overall side effects (Day 0)			
- No	362 (67.5%)	367 (70.2%)	0.3884
- Yes	274 (32.5%)	156 (29.8%)	
- Missing data	20 (3.6% of the total)	33 (5.9% of the total)	
If yes, type of side effect			
- Nausea and vomiting	76 (14.2%)	69 (13.2%)	0.6558
- Faintness	53 (9.9%)	54 (10.3%)	0.8389
- Dizziness	100 (18.7%)	70 (13.4%)	0.0236
- Anxiety	34 (6.3%)	26 (5.0%)	0.3545
- Abdominal pain	11 (2.1%)	11 (2.1%)	>0.9999
Night side effects (Day 0)			
- No	427 (78.3%)	417 (78.1%)	0.9413
- Yes	118 (21.7%)	117 (21.9%)	
- Missing data	11 (1.9% of the total)	22 (3.9% of the total)	
If yes, type of side effect			
- Nausea and vomiting	42 (7.7%)	48 (9.0%)	0.509
- Faintness	25 (4.6%)	28 (5.2%)	0.6736
- Dizziness	67 (12.3%)	55 (10.3%)	0.3365
- Anxiety	30 (5.5%)	26 (4.9%)	0.6818
- Abdominal pain	17 (3.1%)	16 (3.0%)	>0.9999
Night side effects (Day 1)			
- No	415 (76.1%)	395 (72.7%)	0.2111
- Yes	130 (23.9%)	148 (27.3%)	
- Missing data	11 (1.9% of the total)	13 (2 .3% of the total)	
If yes, type of side effect			
- Nausea and vomiting	386 (71.0%)	386 (71.1%)	
- Faintness	30 (5.5%)	35 (6.4%)	0.5253
- Dizziness	62 (11.4%)	76 (14.0%)	0.2031
- Anxiety	28 (5.1%)	25 (4.6%)	0.7785
- Abdominal pain	22 (4.0%)	23 (4.2%)	0.8803
Night side effects (Day 2)			
- No	440 (79.9%)	421 (76.5%)	0.1895
- Yes	111 (20.1%)	129 (23.5%)	
- Missing data	5 (0.9% of the total)	6 (1% of the total)	
If yes, type of side effect			
- Nausea and vomiting	49 (8.9%)	59 (10.7%)	0.313
- Faintness	16 (2.9%)	18 (3.3%)	0.7317
- Dizziness	44 (8.0%)	58 (10.5%)	0.1471
- Anxiety	19 (3.4%)	25 (4.5%)	0.3613
- Abdominal pain	28 (5.1%)	31 (5.6%)	0.6907
Night side effects (Day 3)			
- No	468 (84.9%)	458 (82.8%)	0.1895
- Yes	83 (15.1%)	95 (17.2%)	
- Missing data	5 (0.9% of the total)	3 (0.5% of the total)	0.3682
If yes, type of side effect			
- Nausea and vomiting	30 (5.4%)	34 (6.1%)	0.6995
- Faintness	10 (1.8%)	12 (2.2%)	0.8301
- Dizziness	31 (5.6%)	48 (8.7%)	0.0612
- Anxiety	14 (2.5%)	17 (3.1%)	0.7162
- Abdominal pain	24 (4.4%)	22 (4.0%)	0.7657

Complications

At the last follow-up, the non-opioid group had a trend toward a higher complication rate compared to the opioid group (2.0%, n=11/556 vs. 0.5%, n=3/556; p = 0.0558). This is mainly concerning Cyclops syndrome (36.4%, n=4/11 in the non-opioid group vs. 33.3%, n=1/3 in the opioid group) and knee stiffness (27.3%, n=3/11 in the non-opioid group vs. 0.0%, n=0/3 in the opioid group). There was no difference in the reoperation rate between the two groups (p>0.05) (Table 6).

**Table 6:** Complications between groups. Data are presented as frequencies n (%).

Complications	Non opioid group	Opioid group	p-value
	N=556	N=556	
Overall complication rate			
- No	545 (98.0%)	553 (99.5%)	0.0558
- Yes	11 (2.0%)	3 (0.5%)	
If yes, cause of the complication			
- Cyclops syndrome	4 (36.4%)	1 (33.3%)	0.7527
- Postoperative stiffness	3 (27.3%)	0 (0.0%)	
- Cyclops syndrome and stiffness	4 (36.4%)	2 (66.7%)	
Surgical revision for complication			
- No	2 (18.2%)	0 (0.0%)	0.1438
- Yes	9 (81.8%)	3 (100.0%)	
If yes, type of surgical revision			
- Arthrolysis for Cyclops	5 (55.5%)	1 (33.3%)	0.2692
- Knee debridement	1 (11.1%)	2 (66.7%)	
- Mobilization under GA	3 (33.3%)	0 (0.0%)	

## Discussion

The main finding of this article was that adding morphine to the postoperative protocol is associated with less postoperative pain as well as a lower incidence of dizziness on the first day postoperatively. This indicates the safety of morphine in postoperative protocols. Crucially, while between-group reductions in VAS scores achieved statistical significance at each consecutive postoperative evaluation up to day 3, the mean differences ranged between 0.3 and 0.5 points. Because these absolute variations remain below the conservative MCID threshold of 1.0 to 1.4 established in orthopedic literature, these continuous pain score improvements should not be overinterpreted as representing standalone, clinically meaningful analgesia.

The originality of this study stems from its reliance on an evidence-based protocol that is updated annually. Initially explored by Stein et al. [17], showing no benefit from adding pregabalin to the protocol. Then, subsequent research by Moussa et al. built on these findings, adding dynamic intermittent cryotherapy as an add-on efficient modality to reduce pain [16]. Our current study further evolves this protocol, examining the impact of these regulatory changes on early postoperative pain management.

A noteworthy finding of this study was that early postoperative pain is still higher than figures found in the literature, typically ranging from 2.5 to 5.4 [3,4,17]. In the study by Moussa et al., the authors attributed this difference to the absence of morphine [16]. In our study, the difference persists despite adding morphine, raising questions about possible causes. The main one, in our view, is the technique of collection of VAS outcomes, which is collected online independently in our survey, while it is collected by the surgeon/nurse asking the patient in the literature. This may cause bias driven by the patient underestimating their pain, influenced by the presence of the surgeon. Furthermore, it can be driven by differences in population, culture, and perception of pain between different patients.

Our data demonstrate that the opioid group had a similar side effect profile to the non-opioid protocol while ensuring less dizziness on postoperative day 0. This is in line with existing literature pointing out the side effects of this molecule [18,19]. Furthermore, the increased dizziness in the non-opioid group is likely related to the use of nefopam in this protocol [16,20]. Nefopam is a non-opioid, centrally acting analgesic frequently prescribed in France for acute pain and postoperatively [16,20]. A study by Schulz et al. found that nefopam, although effective, is associated with side effects such as dizziness [20]. The study reported that dizziness is a common side effect, likely due to nefopam’s mechanism of action, which includes inhibition of the reuptake of serotonin, norepinephrine, and dopamine. This aligns with our findings, where the non-opioid group, which included nefopam, experienced a higher incidence of dizziness (18.7% vs. 13.4%, p = 0.0236) compared to the opioid group.

Moreover, when looking at the PASS outcomes, patients in the opioid group tended to reach satisfactory pain levels earlier, with a significant difference emerging from the first postoperative day.

Another interesting finding was the trend toward a higher complication rate in the non-opioid group when it comes to stiffness and Cyclops formation. This may be explained by the increased pain levels in the non-opioid group, which could lead to reduced mobility and compliance with postoperative rehabilitation protocols. Higher pain levels can discourage patients from engaging in necessary physical therapy and exercises, potentially resulting in joint stiffness and scar tissue formation (cyclops lesion). However, this hypothesis is not supported by existing literature, and further studies are needed to establish this correlation.

Although rapid-release oral morphine provided early analgesia, outpatient opioid exposure necessitates rigorous risk-benefit appraisal. In our pathway, oral morphine was prescribed strictly as an as-needed rescue medication rather than a scheduled therapy. However, our registry did not track outpatient prescription refills, exact pill counts consumed, or prolonged opioid dependency beyond the early postoperative period. While short-duration oral opioid protocols under five to seven days carry low documented rates of secondary dependency in acute sports traumatology, the absence of post-discharge consumption data represents a substantial limitation. Consequently, claims regarding overall protocol safety must remain circumspect and restricted solely to immediate acute adverse somatic profiles.

Limitations

This study's retrospective analysis inherently limits its ability to establish causality. However, data were collected prospectively, and there was a low number of missing data. A significant methodological limitation is the multi-agent disparity between both regimens. Because the non-opioid cohort received continuous intravenous nefopam and metoclopramide while the opioid cohort received oral morphine sulfate without nefopam, the study design represents a pragmatic comparison of two multimodal regimens rather than an isolated pharmacological assessment of morphine. Consequently, observed outcomes-including lower early dizziness rates in the opioid cohort-cannot be attributed solely to the presence of morphine, but may instead reflect the absence of nefopam-induced central monoaminergic side effects.

Furthermore, the lack of randomization could lead to selection bias and affect the generalizability of the findings. However, this concern was addressed by a matching technique that was used to reduce potential biases related to patient selection and characteristics.

## Conclusions

An oral morphine-containing multimodal analgesia protocol was associated with modest, statistically significant reductions in early postoperative pain scores and decreased early dizziness compared to an intravenous nefopam-based protocol following primary ACL reconstruction. However, absolute pain reductions on the VAS did not exceed the threshold for minimal clinically important difference, despite a higher proportion of patients reaching acceptable symptom states from day one onward. Given the multi-agent differences between protocols and the retrospective design, these findings reflect an association rather than direct causation, and routine implementation should await prospective randomized controlled trials with formal opioid consumption and dependency monitoring.

## Appendices

Part 1: Patient demographics & background

**Table 7:** Patient demographics & background (completed before surgery).

Variable	Response Options
Age	_______ Years
Sex	Female Male
Sports Practice Level	Sedentary (No sports) Occasional Leisure Regular Leisure Competitive Professional

Part 2: Preoperative functional scores (Completed before surgery)

**Table 8:** Lysholm knee scoring scale.

Knee Condition Category	Patient Response Options (Select one per section)	Score
1. Limp	No limp	5
	Slight or periodical	3
	Severe and constant	0
2. Support	None	5
	Stick or crutch	2
	Weight-bearing impossible	0
3. Locking	No locking and no catching sensations	15
	Catching sensation but no locking	10
	Locking occasionally	6
	Locking frequently	2
	Locked joint on examination	0
4. Instability	Never giving way	25
	Rarely during athletics or other severe exertion	20
	Frequently during athletics or other severe exertion	15
	Occasionally in daily activities	10
	Often in daily activities	5
	Every step	0
5. Pain	None	25
	Inconstant and slight during severe exertion	20
	Marked during severe exertion	15
	Marked on or after walking more than 2 km	10
	Marked on or after walking less than 2 km	5
	Constant	0
6. Swelling	None	10
	On severe exertion	6
	On ordinary exertion	2
	Constant	0
7. Stair-climbing	No problems	10
	Slightly impaired	6
	One step at a time	2
	Impossible	0
8. Squating	No problems	5
	Slightly impaired	4
	Not beyond 90 degrees	2
	Impossible	0
Total Lysholm Score	Sum of sections 1–8 (Maximum Score: 100)	

Tegner activity scale

**Table 9:** Tegner activity scale.

Activity Level	Description of Highest Current Level of Participation	Select One
Level 10	Competitive sports - soccer, football, rugby (national elite)	
Level 9	Competitive sports - soccer, football, rugby (lower divisions), ice hockey, wrestling, gymnastics, basketball	
Level 8	Competitive sports - racquetball or bandy, squash or badminton, track and field athletics (jumping, etc.), down-hill skiing	
Level 7	Competitive sports - tennis, running, motorcars speedway, handball; Recreational sports - soccer, football, rugby, bandy, ice hockey, basketball, squash, racquetball, running	
Level 6	Recreational sports - tennis and badminton, handball, racquetball, down-hill skiing, jogging at least 5 times per week	
Level 5	Work - heavy labor (construction, etc.); Competitive sports - cycling, cross-country skiing; Recreational sports - jogging on uneven ground at least twice weekly	
Level 4	Work - moderately heavy labor (e.g. truck driving, etc.)	
Level 3	Work - light labor (nursing, etc.)	
Level 2	Work - light labor; Walking on uneven ground possible, but impossible to back pack or hike	
Level 1	Work - sedentary (secretarial, etc.)	
Level 0	Sick leave or disability pension because of knee problems	

Part 3: Early postoperative pain & adverse effects diary (Completed on the evening of surgery and the following three days)

Instructions:

Pain Scale (VAS): Rate your pain on a scale from 0 to 10 (0 = No pain at all; 10 = Unbearable pain).

Side Effects: Check all symptoms that you experienced during that specific period.

**Table 10:** Questionnaire of Adverse Effects Experienced

Time Point	Pain Score (VAS 0–10)	Adverse Effects Experienced (Select all that apply)
Day 0: Evening	_______ / 10	Nausea / Vomiting Faintness Dizziness Anxiety Abdominal Pain None
Day 0: Night	_______ / 10	Nausea / Vomiting Faintness Dizziness Anxiety Abdominal Pain None
Day 1: Overall	_______ / 10	Nausea / Vomiting Faintness Dizziness Anxiety Abdominal Pain None
Day 2: Overall	_______ / 10	Nausea / Vomiting Faintness Dizziness Anxiety Abdominal Pain None
Day 3: Overall	_______ / 10	Nausea / Vomiting Faintness Dizziness Anxiety Abdominal Pain None
